# Supplementary material for: Decreasing alpha flow releases task-specific processing paths
Source: Imaging Neurosci (Camb). 2024 Mar 22;2:imag-2-00117. doi: 10.1162/imag_a_00117 (PMC12247566; doi:10.1162/imag_a_00117)
Supplement: Supplementary Material [file imag_a_00117-supp.pdf]

Supplementary Figures

for

Hanna, J., Kim, C., Rampp, S., Buchfelder, M., and N. Müller-Voggel. Decreasing alpha flow releases task-specific processing paths. *Imaging Neuroscience*.

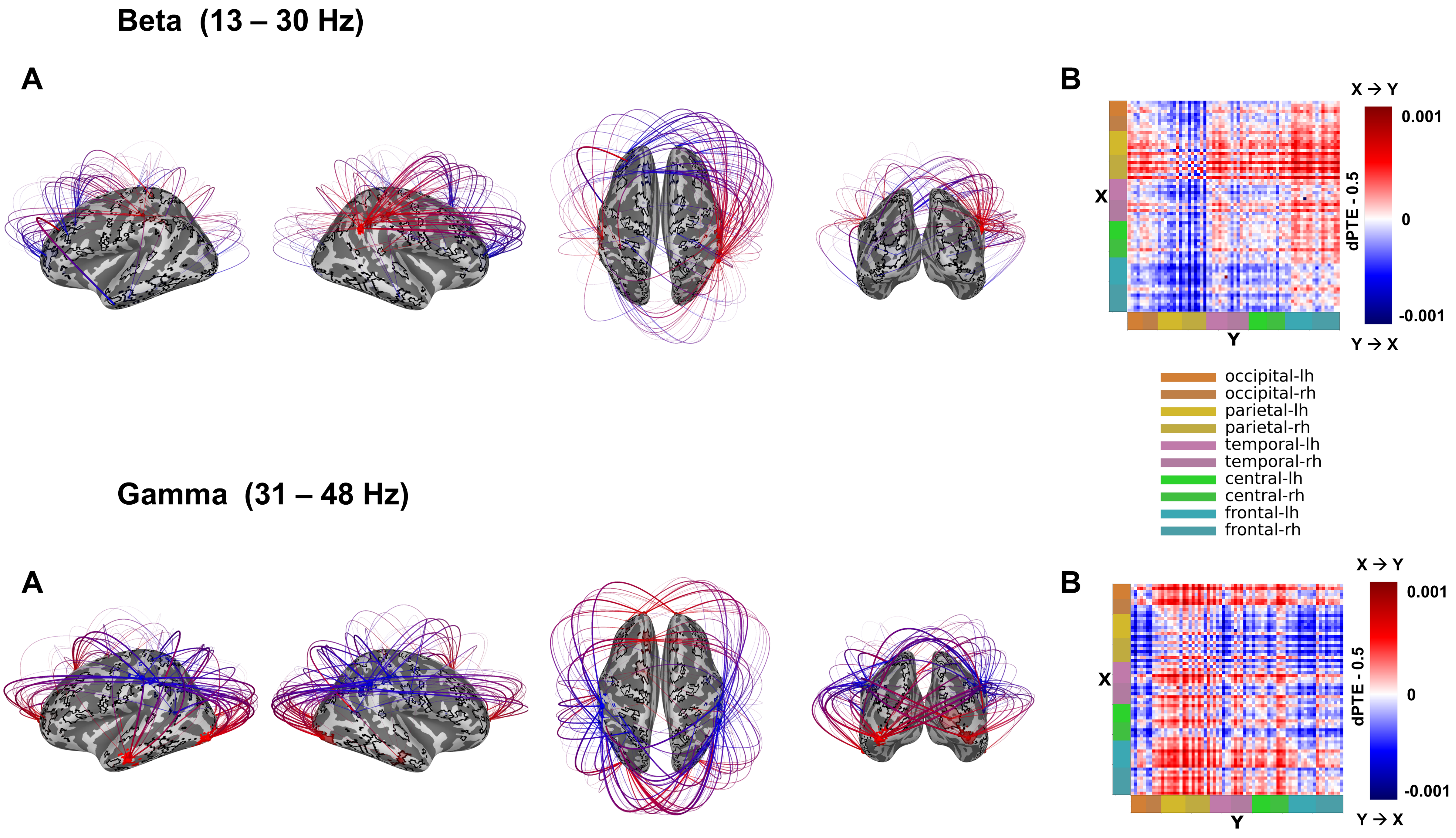

Figure S1: High band resting state directed Phase Transfer Entropy (dPTE) connectivity – Beta (13-30 Hz) and Gamma (31-48 Hz). (**A**) The top 150 strongest connections for each band. Information movement from source to destination is indicated by red to blue. (**B**) Connectivity matrix for all connections at rest. Values represent zero-centered dPTE values (subtracting 0.5) for better readability of direction. Lines of connections are grouped into colors by lobe and hemisphere (see Fig. 1 in main manuscript).

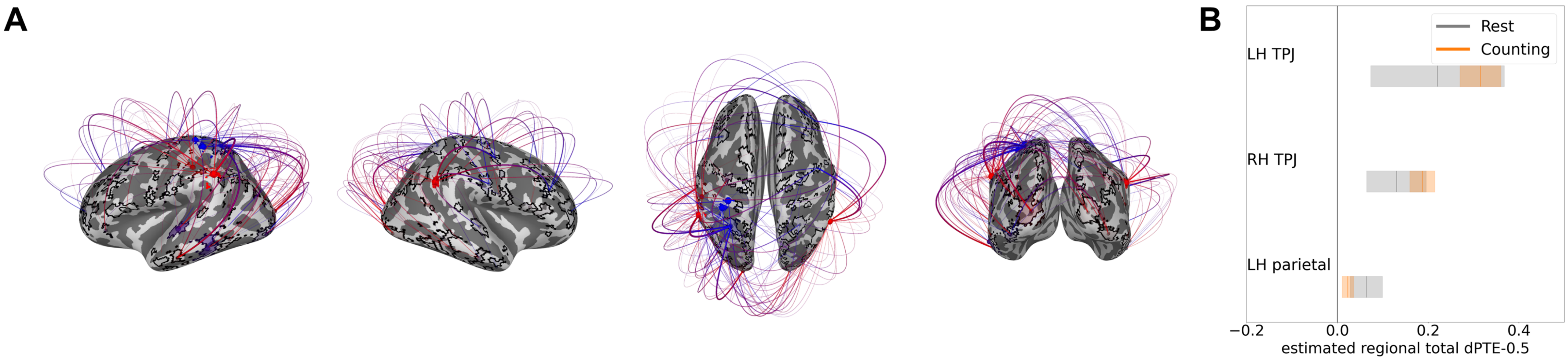

Figure S2: High Alpha band (10-13 Hz) dPTE changes from resting state during the backwards counting task (internal attention). (**A**) Top changes from resting state during backwards counting. Connection lines represent LME model parameters of this condition and need to be interpreted with reference to the resting state values in Fig. 3A–B (main manuscript). (**B**) Summary of changes for most prominently affected hubs in (A). Bars display summed zero-centered dPTE values for hub area to all other regions with 95% CIs, comparing resting state (grey) and backwards counting (light orange).
